# A Global Challenge: Sustainability of Submicrometer PEO and PVP Fiber Production

**DOI:** 10.1002/gch2.202300152

**Published:** 2023-08-31

**Authors:** Manul Amarakoon, Shervanthi Homer‐Vanniasinkam, Mohan Edirisinghe

**Affiliations:** ^1^ Department of Mechanical Engineering University College London Torrington Place London WC1E 7JE UK

**Keywords:** energy consumption, green engineering, manufacture, polymeric fiber, sustainability

## Abstract

The field of submicrometer polymeric production currently has a predominant research focus on morphology and application. In comparison, the sustainability of the manufacture of submicrometer polymeric fibers, specifically the energy efficiency, is less explored. The principles of Green Chemistry and Green Engineering outline frameworks for the manufacture of “greener” products, where the most significant principles in the two frameworks are shown to be centered on energy efficiency, material wastage, and the use of non‐hazardous materials. This study examines the power consumption during the production of Polyethylene oxide (PEO) and Polyvinylpyrrolidone (PVP) submicrometer fibers under magnitudes of the key forming parameters to generate fibers via pressure spinning. The energy consumption, along with the fiber diameter, and production rate during the manufacture of fibers is predominantly attributed to the characteristics of polymeric solutions utilized.

## Graphical abstract

Polymeric fibers are key engineering materials, however, they present environmental challenges. The study investigates sustainable production using Polyethylene oxide (PEO) and Polyvinylpyrrolidone (PVP), emphasizing green engineering principles. PVP exhibited better energy efficiency and higher production rates, however PEO generated smaller diameter fibers, with optimal control achieved at 35% concentration and 0.2 MPa pressure for both polymers produced via pressurized gyration.

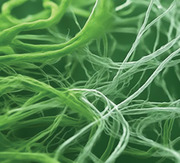

## 1 Introduction

Polymeric fibers are used in many applications, for instance, in increasing quantities in the biomedical industry due to their resemblance with the extracellular matrix and along with their versatility.^[^ ^1^ ^]^ Methods of obtaining polymeric fibers are diverse, where electrospinning is the most common method currently used to generate fibrous submicrometer biomaterial.^[^ ^1^, ^2^ ^]^ There have been several attempts to enhance the sustainability of polymeric fibers, such as the use of natural polymers and biodegradable polymers to minimize environmental impact. However, these do not usually account for potential environmental effects during the manufacturing stage of polymeric submicrometer fibers. The subject of sustainability has advanced to a stage where virtually every field of manufacturing has sustainability initiatives.^[^ ^3^ ^]^ Sustainability is also seen as a business approach, which enhances value for an organization whilst improving its environmental impact, along with the public and economy.^[^ ^4^ ^]^ Businesses have also increasingly acknowledged the merit of sustainability, such as in elevated publicity along with reduced manufacturing cost, energy, hazards, and risk.^[^ ^5^ ^]^ This can also be reflected in the field of biomaterials and industries that make use of submicrometer polymeric fibers such as the healthcare sector. There is a strong focus on the use of more sustainable materials in the production of polymeric submicrometer fibers, such as the use of biomaterials and non‐hazardous solvents. However, there seems to be a lack of emphasis on sustainability during the actual manufacture of submicrometer polymeric fibers.

Sustainable manufacturing is the making of products via economically sound methods which promote minimal environmental impacts, energy efficiency, and the use of natural resources. Sustainable manufacturing also increases employee, public, and product safety, which subsequently increases operational efficiency, brand/product reputation, and competitive advantage.^[^ ^6^ ^]^ In the case of polymeric nanofiber manufacture, the energy efficiency and safety factor are generally less discussed. Methods such as phase separation and self‐assembly can be inefficient in terms of energy consumption, whilst there are safety concerns such as the use of large voltages in electrospinning.^[^ ^7^ ^]^ In comparison, pressurized gyration is an energy‐efficient and safer process of producing polymeric fibers.^[^ ^8^ ^]^ This is due to its simplicity and production efficiency in comparison to some of the other popular polymeric nanofiber production methods, such as phase separation and template synthesis.^[^ ^8^ ^]^ The application of sustainable manufacturing has the potential to amplify an already sustainable process to produce the “greenest” submicrometer polymeric fibers.

**Figure 1:**
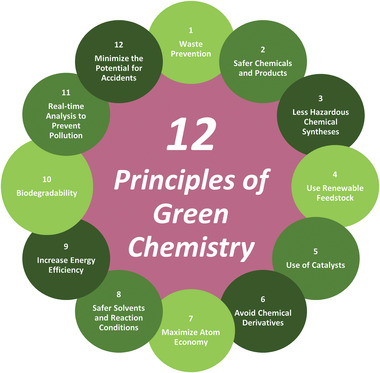
The 12 principles of Green Chemistry.^[^ ^9^ ^]^

The evolution of sustainable technologies has been tackled in multiple approaches, where utilizing the principles of Green Chemistry is an advantageous approach, especially when dealing with polymer‐based products.^[^ ^9^ ^]^ As a developed and highly adaptable field, the polymer industry plays an important role, as polymers are ubiquitous in modern society. However, drawbacks such as the large‐scale use of petroleum‐based raw materials and vast quantities of reagents that are of environmental threat, along with the build‐up of polymeric matter in the environment, bestows engineers and researchers the liability to re‐evaluate the manufacture of polymers with regards to the 12 principles of Green Chemistry (**Figure** **1**).^[^ ^9^ ^]^

**Figure 2:**
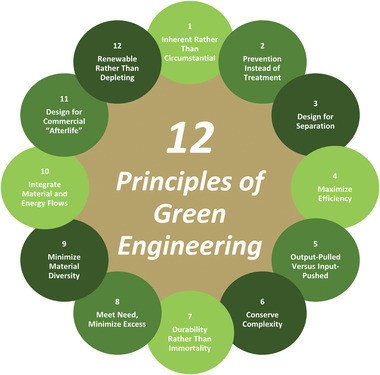
The 12 principles of Green Engineering.^[^ ^10^ ^]^

Overall, the 12 principles propose the design of chemical reactions and syntheses to promote safety, minimize waste and optimize energy efficiency. Arguably, principles 2, 3, 4, 8, and 9 can be deduced to have the capability to generate the most significant environmental benefit.^[^ ^9^ ^]^ Principle 2, 3, and 8 emphasizes the use of non‐toxic alternatives and reaction conditions such as the use of non‐toxic solvents in controlled conditions in the production of polymer products. Principle 4 is an extensively researched area especially in the making of polymeric fibers, regardless of constraints in the supply of renewable feedstock for polymeric fiber. Principle 9 promotes energy efficiency which is currently an aspect in the production of polymeric fibers that is not as extensively researched. Similar to the 12 Principles of Green Chemistry, the 12 principles of Green Engineering (**Figure** **2**) were determined to allow engineers to integrate features of sustainability in all areas of a project in a systematically comprehensive procedure.^[^ ^10^ ^]^

Green engineering involves the development, marketing, and utilization of methods and goods with the aim of decreasing pollution, fostering sustainability, and mitigating potential harm to human health and the environment, all while maintaining economic feasibility and effectiveness.^[^ ^10^ ^]^ The main principles of Green Engineering promotes sustainability via design to enhance efficiency, simplicity, material efficiency, and renewability. Seemingly, many of the principles of Green Chemistry and Green Engineering are integrated, where energy efficiency, minimization of wastage, and the use of sustainable materials are promoted. The application of the principle of Green Chemistry and Green Engineering in the production of polymeric submicrometer fibers is an appropriate foundation to promote sustainability. The aim of this study is to evaluate how Green Chemistry and Green Engineering can be further incorporated into the pressurized gyration method to produce polymeric fiber, primarily by promoting energy efficiency as this reflects the principles of both Green Chemistry and Green Engineering. In the case of pressurized gyration, which was evaluated to be an energy‐efficient process in comparison to other polymeric submicrometer manufacturing methods, an approach to evaluate its energy efficiency is to study various magnitudes of parameters that affect the fiber output with a focus on its energy consumption.^[^ ^8^ ^]^ This approach is used in this investigation using Polyethylene oxide (PEO) and Polyvinylpyrrolidone (PVP) to produce fibers via pressurized gyration. Both PEO and PVP are polymers with very low toxicity in comparison to other synthetic polymers and water‐soluble which as a consequence compliments the principles of Green Chemistry.^[^ ^11^ ^]^ Natural polymers are more “greener” however, synthetic polymers are functionally superior to natural polymers. Therefore, PEO and PVP was selected as a middle ground for this study as the polymers are both functional and less hazardous. Polymeric fibers with smaller diameters are idealized in many applications as they allow for enhanced surface‐to‐volume ratio, low density, high porosity, and high flexibility.^[^ ^12^ ^]^ For instance, in filtration‐related applications a higher surface‐to‐volume ratio promotes particle capture of a smaller scale and hence increases filtration efficiency. The paper investigates the energy consumption during the production of submicrometer polymeric PEO and PVP polymeric fibers using pressure spinning, which is a leading contender in the quest for reducing environmental impact of polymer fiber manufacture.^[^ ^8^ ^]^ Process control parameters were varied and the resulting energy consumption along with other output properties are calculated. Statistical analysis was used to recognize the main factors that influence energy consumption. The necessity of a full life cycle analysis to achieve optimal sustainability is proposed.

### 1.1 Pressurized Gyration

**Figure 3:**
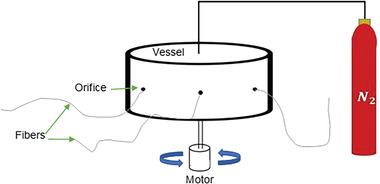
Schematic of the pressurized gyration method.

The manufacturing process of pressurized gyration is predominantly undertaken at ambient temperatures. The method is less complex and more straightforward in comparison to many other submicrometer polymeric fiber‐manufacturing methods.^[^ ^13^ ^]^ The simplest model of the pressurized gyration method used in this study has polymeric solution placed inside a rotating vessel before being spun at high speeds along with the application of pressure into the vessel if necessary. The centrifugal forces generated from the spinning of the vessel along with the additional pressure difference compels the solution to jet out through orifices in thin streams and the solvent to subsequently evaporate in the process leaving behind polymer fibers. The fibers are gathered in a collector around the vessel which is positioned at a selected distance from the wall of the vessel. A schematic of this method is shown in **Figure** **3**, where a motor connected to a rotating vessel holding polymeric solution can be supplemented with applied pressure to extrude fibers out of the orifices of the vessel.

Various process control parameters affect the overall result of the substance that is jetted out from the orifices of the vessel.^[^ ^14^ ^]^ The correct magnitudes applied to each parameter determines if any fibers are formed along with the properties of the fibers generated. Understanding how the magnitudes affect fiber production in terms of green manufacturing can be crucial to produce the “greenest” product.

Changes in parameters will affect the properties of fiber such as its diameter and morphology, along with other properties such as the internal structure of the fiber. At present, there has been a strong focus on research on these physical and chemical properties such as the diameter and morphology of submicrometer polymeric fibers along with their applications. However, the sustainability aspect such as the energy consumption to produce these fibers are less discussed.

**Figure 4:**
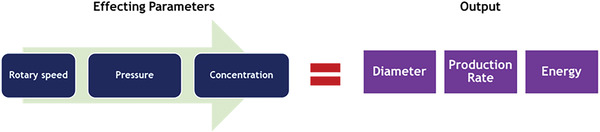
The effecting and output parameters evaluated in this study.

Previous studies indicate that the rotation of the vessel, along with the pressure and concentration of the polymeric solution are the primary parameters that contribute to the forming of fibers in pressurized gyration.^[^ ^14^ ^]^ In the case of infusion‐based gyration methods, the infusion rate also plays a significant part in the characteristics of resulting fibers.^[^ ^15^ ^]^ This is also the case in pressurized melt gyration where the polymer is melted (rather than in solution, avoiding solvents) and hence the temperature applied is another important parameter. Other parameters such as collector distance and environmental conditions dictate the properties of the fibers. For instance, relative humidity plays a major role in the making of fibers that use a water‐based solvent or a water‐soluble polymer due to the low vapor pressure of water.^[^ ^16^ ^]^ However, this along with temperature can be maintained. To understand this, a study was undertaken with a focus on how the variation of rotary speed, pressure, and concentration affects energy consumption, along with fiber diameter and production rate during the pressurized gyration process as portrayed in **Figure** **4**. The morphology of the fibers produce, specifically the fiber diameter is analyzed to maintain the relevance of the produced fibers for applications, whereas the production rate is evaluated to understand material efficiency.

## 2 Experimental Section

### 2.1 Materials

Polyvinylpyrrolidone (PVP, Mw ≈ 1 300 000, CAS: 9003‐39‐8), and polyethylene oxide (PEO, Mw ≈ 200 000, CAS: 25322‐68‐3) were obtained from Sigma‐Aldrich (Gillingham, UK) and used as received. The solvent used was distilled water.

### 2.2 Solution Preparation and Characterization

PEO and PVP were dissolved separately in distilled water to produce polymer solutions of concentrations of 30, 35, 40, and 50 wt.%. Homogeneity was achieved by magnetically stirring all eight solutions for 24 h at the ambient temperature (°C) and relative humidity (%). The PEO‐H2O and PVP‐H2O solution viscosities used in this study were characterized using a calibrated Brookfield Viscosity‐meter where the results are displayed in **Table** **1**.

**Table 1:** Experimental results of solutions.

Polymer‐Solvent	% [w/v]	Viscosity [mPa s]	Pressure [MPa]	Speed [RPM]	Power [W]	Fiber mass [g]	Spin time [s]	Production rate [g hr^−1^]	Diameter [µm]	Energy [J]
PEO‐H_2_O	30	9418	0	12 000	30.7	0.057	35	5.9	0.615 ± 204	1075
PEO‐H_2_O	30	9418	0.1	11 400	35.7	0.088	30	10.6	0.387 ± 119	1071
PEO‐H_2_O	30	9418	0.2	10 900	38	0.089	30	10.7	0.316 ± 132	1140
PEO‐H_2_O	35	19 576	0	12 000	31	0.079	45	6.32	0.627 ± 231	1395
PEO‐H_2_O	35	19 576	0.1	11 400	35.1	0.126	40	11.34	0.371 ± 132	1404
PEO‐H_2_O	35	19 576	0.2	10 900	37.1	0.138	40	12.42	0.321 ± 127	1484
PEO‐H_2_O	40	29 544	0	12 000	32	0.148	60	8.88	1.215 ± 0.427	1920
PEO‐H_2_O	40	29 544	0.1	11 400	37	0.174	50	12.528	0.631 ± 0.292	1850
PEO‐H_2_O	40	29 544	0.2	10 900	38	0.176	50	12.672	0.578 ± 0.134	1900
PEO‐H_2_O	50	59 387	0	12 000	32	0.187	75	8.976	2.385 ± 0.714	2400
PEO‐H_2_O	50	59 387	0.1	11 400	37	0.226	60	13.56	1.089 ± 0.455	2220
PEO‐H_2_O	50	59 387	0.2	10 900	38	0.233	60	13.98	0.889 ± 0.389	2280
PVP‐H_2_O	30	3879	0	12 000	31	0.019	10	6.84	0.640 ± 0.286	310
PVP‐H_2_O	30	3879	0.1	11 400	35	0.031	8	13.95	0.455 ± 0.145	280
PVP‐H_2_O	30	3879	0.2	10 900	37	0.037	8	16.65	0.451 ± 0.159	296
PVP‐H_2_O	35	4255	0	12 000	32	0.021	10	7.56	0.704 ± 0.266	320
PVP‐H_2_O	35	4255	0.1	11 400	37	0.044	8	19.8	0.532 ± 0.205	296
PVP‐H_2_O	35	4255	0.2	10 900	38	0.049	8	22.05	0.501 ± 0.214	304
PVP‐H_2_O	40	7483	0	12 000	32	0.075	15	18	1.869 ± 0.780	480
PVP‐H_2_O	40	7483	0.1	11 400	37	0.089	12	26.7	0.941 ± 0.518	444
PVP‐H_2_O	40	7483	0.2	10 900	38	0.093	12	27.9	0.627 ± 0.197	456
PVP‐H_2_O	50	14982	0	12000	32	0.087	15	20.88	2.642 ± 0.833	480
PVP‐H_2_O	50	14982	0.1	11400	37	0.117	13	32.04	1.232 ± 0.652	481
PVP‐H_2_O	50	14982	0.2	10900	38	0.121	13	33.50769	1.086 ± 0.503	494

### 2.3 Fiber Production and Characterization

**Figure 5:**
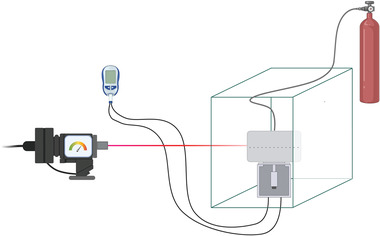
Experimental setup.

The pressurized gyration device shown in Figure 3 was set up with a laser tachometer aimed at the rotating vessel, a power meter connected to the motor along with a video recorder which was set up to obtain real‐time readings of the rotary speed of the vessel and power usage during each experiment (**Figure** **5**). Each concentration of PVP and PEO solution utilized in this experiment was subjected to an applied pressure of 0 (no gas flow), 0.1, and 0.2 MPa, which makes a total of 24 experimental samples of 4 ml each. The video recorder was used during each experiment to obtain real‐time footage of the tachometer and power meter readings whilst evaluating the time the vessel was spun during the extrusion of fibers from the orifices of the vessel.

The collector distance was set at 100 mm where the laser tachometer readings show that the vessel reached average rotatory speeds of 12 000, 11 400, and 10 900 RPM at applied pressures of 0, 0.1, and 0.2 MPa, respectively.

The production rate of the resulting fibers were calculated by measuring the mass of the fibers produced using a very sensitive scale and evaluating the time the fibers took to spin by reviewing video footage. The fiber diameter was evaluated using a scanning electron microscope (SEM). The micrographs acquired via SEM imaging were analyzed using Image J software to obtain the average fiber diameter. Energy consumption was calculated using a power meter connected to the power socket which displays the power drawn by the motor when used. The time taken to spin the fibers along with the average power meter reading was used to estimate the energy consumption.

## 3 Results

A notable change in the rotary speed of the vessel was not identified with the use of the different concentrations of PEO and PVP. On average, under the same effecting parameters (RPM, concentration, and Pressure), the power consumption was very similar, where an average reading of 31.5 W is shown at 0 MPa for each sample, which increased by 5 W to an average of 36.4 W when a pressure of 0.1 MPa is applied and an average of 37.8 W at an applied pressure of 0.2 MPa. However, the actual power readings for each sample is utilized in this study as shown in Table 1 to maintain the accuracy of energy consumption findings.

Under the same magnitudes of affecting parameters, the energy consumption to produce PVP fibers is shown to be lower than the energy consumption to produce PEO as shown in Table 1. This is primarily due to the lower spin time required to produce PVP fibers using the same volume of polymeric solution as PEO, where the spin time is attributed to the viscosity of the solutions along with the applied pressure magnitudes. The production rate of PVP fibers was evaluated to be higher than PEO fibers under the same magnitudes of effecting parameters, which is attributed to the molecular weight of PVP (Mw ≈ 1300000) used in the study being higher than that of PEO (Mw ≈ 200000). This is due to the spin time of PVP fibers being significantly lower than that of PEO fibers using the same volume of polymeric solution results in a higher production rate for producing PVP fibers.

**Figure 6:**
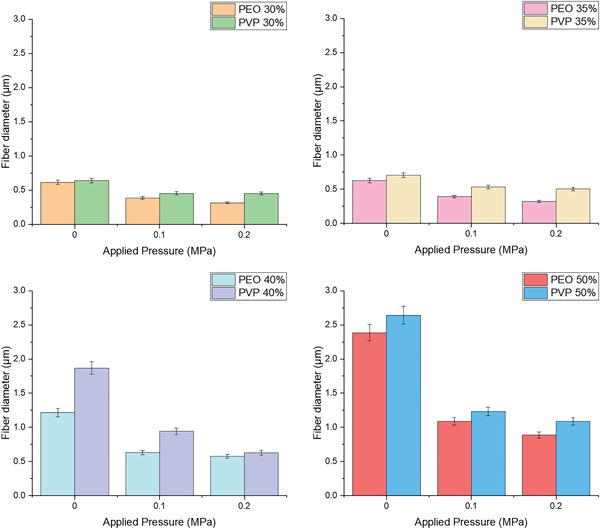
The effects of applied pressure magnitude on fiber diameter for PEO‐H_2_O and PVP‐H_2_O samples (where the error bars represent the standard deviation).

**Figure** **6** illustrates that the application of pressure decreased the fiber diameter of both PEO and PVP at all concentrations. However, this reduction is less significant when the applied pressure magnitude is increased from 0.1 to 0.2 MPa, in comparison to the reduction of fiber diameter caused by the application of a pressure magnitude of 0.1 MPa from no pressure.

**Figure 7:**
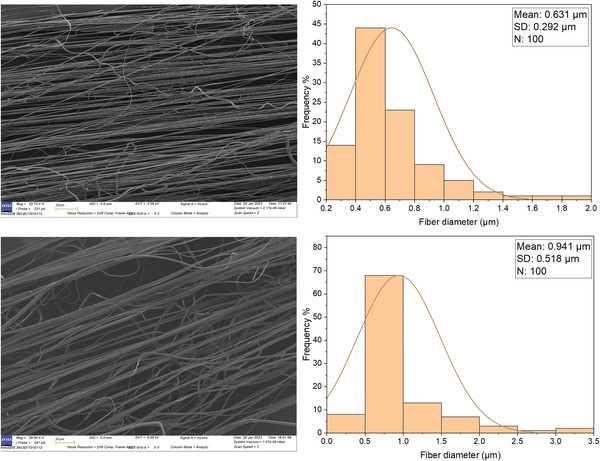
Scanning Electron Microscope Image of PEO 40% (top left) and PVP 40% fibers (bottom left), along with corresponding fiber distribution graphs (right) produced under an applied pressure of 0.1 MPa (where the error bars represent the standard deviation).

PEO fibers were shown to have a relatively smaller diameter in comparison to PVP fibers under the same magnitudes of the three effecting parameters taken into consideration in this study (rotary speed, pressure, and the concentration of the polymeric solution). For instance, at a concentration 40% under an applied pressure of 0.1 MPa, PEO fibers resulted in an average diameter of 0.631 µm, whereas PVP fibers resulted in an average diameter of 0.941 µm as depicted in **Figure** **7**. The larger diameter of PVP fibers is attributed to the higher molecular weight of the polymer used, which affects the ability of the polymer solution to flow and form thin fibers.

The results in Table 1 show that for all four concentrations of PEO and PVP, the difference in the mass of fibers collected was significantly less than the difference in the mass of fibers collected at 0 and 0.1 MPa. Regardless, a small increase in the mass of fibers collected with the application of pressure from 0.1 to 0.2 MPa is still seen. The spin time taken for fibers to be extruded also showed that the application of a pressure of 0.2 MPa did not show a noticeable difference in comparison to an applied pressure of 0.1 MPa for both PEO and PVP at all concentrations.

**Figure 8:**
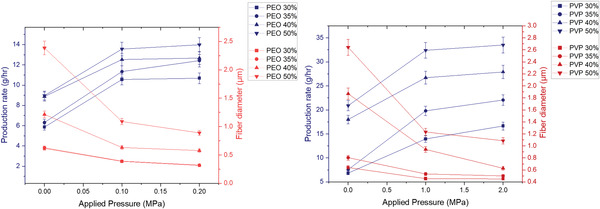
The effects of applied pressure magnitudes on fiber production rate (blue) and fiber diameter (red) for PVP‐H_2_O (right) and PEO‐H_2_O (left) samples (where the error bars represent the standard deviation).

**Figure** **8** indicates that both PVP and PEO fibers experienced a decrease in fiber diameter along with an increase in production rate under increasing pressure magnitudes. However, the increase in production rate is less significant from 0.1 to 0.2 MPa in comparison to the increase from 0 to 0.1 MPa as seen in Figure 8. In general, the production rate for PVP fibers was lower than that of PEO fibers, particularly at higher concentrations and pressures. For instance, at a concentration of 40%, the PEO fibers had a production rate of 12.5 g hr^−1^ at 0.1 MPa pressure, while the PVP fibers had a production rate of 26.7 g hr^−1^ under the same conditions. This is attributed to the differences in the solution properties of the two polymers as seen in Table 1, which can affect their ability to flow and form fibers under pressure.

**Figure 9:**
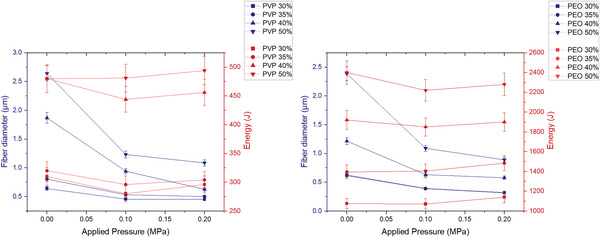
The effects of applied pressure magnitudes on fiber diameter (blue) and energy consumption (red) for PVP‐H_2_O (left) and PEO‐H_2_O (right) samples (where the error bars represent the standard deviation).

Although the magnitudes of the power consumption were comparable at the same magnitudes of the effecting parameters, a notable difference in the total energy to produce both PVP and PEO fibers is comprehended (**Figure** **9**). The energy consumption is directly proportional to the time taken for each 4 ml sample to produce fibers. PEO used a significantly longer time in comparison to PVP to produce fibers at the same concentrations and magnitudes of pressures as seen in Table 1. Hence, the energy consumption for PVP fibers was lower than that of PEO fibers at all concentrations and pressures tested. This is due to differences in the rheological properties of the two polymer solutions as well as the differences in their ability to form fibers under pressure.

**Figure 10:**
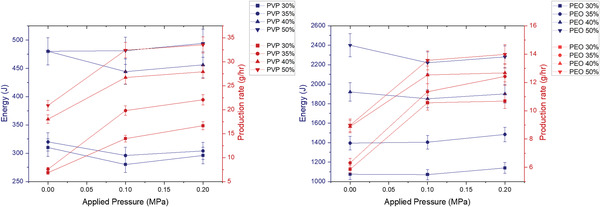
The effects of applied pressure magnitudes on fiber production rate (red) and energy consumption (blue) for PVP‐H_2_O (left) and PEO‐H_2_O (right) samples (where the error bars represent the standard deviation).

The increase of applied pressure from 0.1 to 0.2 MPa does not show a better performance in energy consumption to produce PEO and PVP fibers in comparison to an increase in applied pressure from 0 to 0.1 MPa. This is due to the result of an application of pressure of 0.2 MPa which did not show a perceptible improvement in the time taken to form fibers but increased the power consumption to an average of 37.4 W across all samples as seen in Table 1. The experimental video footage did not identify a significant decrease in spin time due to the increase in applied pressure from 0.1 to 0.2 MPa. Hence the energy consumption was evaluated to be higher at an applied pressure magnitude of 0.2 MPa in comparison to an applied 0.1 MPa for most of the samples in the study as elucidated in **Figure** **10**.

Overall, it can be established that to result in optimum efficiency to produce PVP and PEO fibers, utilizing a pressure magnitude of 0.1 MPa is ideal taking into consideration the magnitudes of affecting parameters used in this study. Optimum efficiency considers the highest production rate and lowest fiber diameter (depending on applications) using the lowest energy consumption.

**Figure 11:**
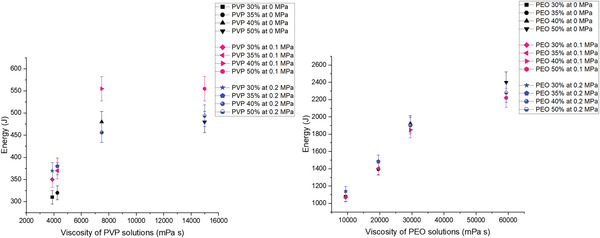
Energy consumption to produce fibers at viscosities associated with each concentration of PVP (left) and PEO (right) (where the error bars represent the standard deviation).

**Figure** **11** shows that as the viscosity of a specific polymer solution increases, the energy consumption required for the pressurized gyration process also tends to increase. This is particularly evident when comparing the data for PEO 30%, 35%, 40%, and 50%, where the highest viscosity polymer solution (PEO 50%) required the most energy to produce fibers with the desired characteristics. One reason for this trend is that higher viscosity solutions require higher pressures and speeds to achieve the desired fiber diameter and production rate, which in turn require more energy.^[^ ^17^ ^]^ Furthermore, highly viscous solutions also require more power to overcome the increased resistance to flow through the spinning equipment.

## 4 Discussion

Overall, the results show that PVP performed better than PEO in terms of energy efficiency and production rate under the same magnitudes of effecting parameters. However, PEO was superior in terms of obtaining lower fiber dimeters under the same magnitudes of effecting parameters. Hence, it is inconclusive if PEO or PVP is preferable over the other to produce fibers via pressurized gyration considering both efficiency and application.

With respects to the resulting production rate, energy consumption and fiber diameter due to the range of magnitudes of the affecting parameters in this study, it can be judged that both PEO and PVP performed best under an applied pressure of 0.2 MPA at a concentration of 35%. PEO fibers produced under these magnitudes of the affecting parameters the fiber diameter were only 5 nm larger from value of the smallest PEO fiber diameters produced in this study, whereas the production rate was only 1.5 g hr^−1^ less than highest production reached at a value of 13.98 g hr^−1^ at a concentration of 50% and applied pressure of 0.2 MPa. The energy consumption under for PEO 35% at an applied pressure of 0.2 MPa was calculated to be 1484 J which is less than the median value for energy to produce PEO fibers in this study. In the case of PVP fibers at an applied pressure of 0.2 MPa and concentration of 35%, the diameter of the fibers and the energy to produce them were only 50 nm and 9 J respectively, more than the value of the fiber produced at a concentration of 30% under 0.2 MPa. The production rate was calculated to be 22.05 g hr^−1^ was over the median value for PVP fibers.

It has been concluded in previous literature that the rotary speed of the gyrating vessel along with the applied pressure are the primary parameters that dictate the morphology such as the fiber diameter of resulting fibers in the pressurized gyration method.^[^ ^14^ ^]^ There has been a focus on how various rotational speeds affect fibers when using pressurized gyration, where higher speeds are associated with lower diameters of fibers which are idealized for many applications due to the resulting higher surface area to volume ratio.^[^ ^18^ ^]^ High rotational speeds supplemented with high applied pressure aid the extrusion process of fibers by producing higher centrifugal force along with force due to applied pressure to obtain diameters in the nanoscale. However, higher speeds may not be as energy efficient, due to the requirements of motors to draw in larger currents to reach such speeds and hence larger output power magnitudes. In comparison, this study shows that increasing the pressure does not drastically increase the power consumption of the motor. However, it was also summarized that increasing the pressure from 0.1 to 0.2 MPa did not result in a significant decrease in fiber diameter and increase in production rate, in comparison to increasing the pressure from 0 to 0.1 MPa. Regardless, the effects of higher magnitudes of applied pressure over 0.2 MPa can be explored and may be an effective approach to obtain fibers of lower diameters and higher production rates more energy efficiently.

Furthermore, the results of this study proves that the viscosity of the polymeric solution is directly proportional to energy consumption to form fibers using the pressurized gyration method. Nevertheless, there is a need to also consider the surface tension of the solvent in the polymeric solution as gas pressure acting as the primary driving force can cause a rapid loss of solvent from the polymeric solution.^[^ ^19^ ^]^ However, this study used water which is considered the “greenest” solvent. The surface tension of water is ≈72.8 mN m^−1^at room temperature which is high in comparison to other commonly used solvents such as acetone which has a surface tension of 24.5 mN m^−1^.^[^ ^20^ ^]^ Hence, the effects of applied gas pressure magnitudes and the rapid loss of solvents when using water as the solvent is comparatively insignificant.

During the study, it was observed that increasing the pressure immediately caused solution and polymers to spray out of the orifices rather than in fiber form, in comparison to applying pressure more gradually to the required magnitude once the rotary speed of the vessel is about to reach its critical speed for fiber formation. To abide by the principles of green engineering, more timed control of the application of pressure promotes less wastage of solution to optimize fiber yield as an immediate application of applied pressure causes the polymeric solution to spray or jet out of the orifices. Minimal wastage of polymeric solution subsequently increases the production rate of the system as the mass of fibers formed will be optimal. The experimental setup for pressurized gyration used in this research did not include infusion of the polymeric solution. However, infusion‐based gyration methods such as pressure coupled‐infusion gyration can promote less wastage, optimize production rates, and control fiber diameter.^[^ ^21^ ^]^ This can be further improved by adjusting the magnitude of infusion concerning parameters such as the rotational speed of the vessel and the concentration of polymeric solution utilized. Identifying exactly when a specific polymeric solution needs to be within the vessel once the motor has started (once the vessel has reached its minimum critical rotational speed) and having the polymeric solution enter the vessel at this precise time can ensure minimal wastage. It can also be estimated how long the motor is required to run for solutions to be infused into the vessel and converted into fibers. Using the volume of the solution along with the flow rate of infusion, the exact time the motor is required to stop to avoid unnecessary use of the motor (when there is no solution in the vessel) can conserve energy.

It is comprehended that the overall load on the motor dictates the power drawn by the motor. This is not only seen in the application of pressure but also when the motor is turned on when it is loaded with the vessel and when the motor is turned on when unloaded. The power drawn when the motor is turned when unloaded is equal to the power rating for the Nichibo motor used in this study, which is 21.2 W.^[^ ^22^ ^]^ When the motor is run under load, the power drawn will be higher than the rated power of the motor. However, lesser loads on the motor will show that the power drawn will be closer to the rated power of the motor.

In general, metal vessels are popularly used for pressurized gyration systems. However, plastic vessels have also been used, where the plastic vessel has less load on the motor in comparison to the heavier metal vessel which therefore promotes better energy efficiency.^[^ ^23^ ^]^ However, the plastic vessel cannot be used with solvents that may cause undesirable interactions with plastic surfaces. Carbon fiber can be a suitable material substitute as carbon fiber (in its rigid form) is usually used simultaneously with a polymer matrix to produce lightweight components. The epoxy resin used in carbon fiber makes the material chemically resistant to most alcohols, acids, and other chemical compounds.^[^ ^24^ ^]^ Along with this, the low thermal expansion of carbon fiber is ideal for vessels used in gyration‐based polymeric fiber manufacturing methods as they can withstand extreme heat.

It can be argued that the design of current submicrometer polymeric fiber manufacturing methods has been focused on quality and safety specifications. Consideration of green engineering principles and green chemistry principles in the design of polymeric fiber manufacturing methods can be utilized to enhance or consider environmental, social, and economic factors.^[^ ^10^ ^]^ It is beneficial to visualize these principles as parameters where the application of one parameter may enhance one or more other principles of green engineering and/or green chemistry. Regardless, two of the most important concepts that designers are pushed to endeavor are considering the lifecycle and the first principle of green engineering, which promotes the minimal use of hazardous materials and energy obtained from hazardous means.^[^ ^10^ ^]^

A life cycle analysis (LCA) is a method that can be used to evaluate environmental impacts such as energy consumption at all stages of polymeric fiber. The materials and energy inputs at every section of the life cycle of a specific product and process fully captures their life cycle. If a product is environmentally friendly but is made using hazardous or non‐renewable materials, the impacts are merely transferred to another part of the overall life cycle. Polymer and solvent selection are vital when considering the lifecycle. In the case of pressurized gyration, regardless of the method's energy efficiency in comparison to other processes, if the extraction and manufacture of certain polymers and solvents offset any energy savings, there is no net sustainable advantage. This study evaluated how the environmental impact can be improved in terms of energy used in the “manufacture” stage of the LCA of PEO and PVP polymeric nanofibers produced using pressurized gyration.

Synthetic polymers are produced via polymerization, which is an exothermic process and is derived from fossil fuels. Therefore, it is useful to not only consider the functional and safety aspect of polymers but also the life cycle of the polymer material itself. Similarly, for the solvent used (distilled water), the distillation process of water requires the liquid to be heated until evaporation, along with nitrogen gas compression methods and energy to produce various concentrations of polymeric solutions can compromise overall energy efficiency. However, natural water is likely to suffice equally well in case commercial manufacturing is pursued. This study also proved that highly viscous polymeric solutions consumed more energy to produce polymeric fibers. This is also the case during the preparation of polymeric solutions of the same volume, where the high viscous solutions used in this study require more time to reach homogeneity on the magnetic stirrer. Hence, it is necessary to undertake a full life cycle analysis, including energy consumption during the application or use of polymeric fibers along with the distribution and end of life. However, the scope for this is too wide when considering the range of polymers, solvents, and the number of applications polymeric fibers are utilized for. An approach may be to identify the most popular application and most popular polymer used to produce fibers for this application and evaluate its life cycle considering the most efficient method of manufacture.

## 5 Conclusion

Based on the findings of this study, there were notable differences between the performance of PEO and PVP used in the pressurized gyration process. Overall, these differences highlight the importance of selecting the appropriate polymer for a given application in the pressurized gyration manufacturing. Both PVP and PEO performed best under an applied pressure magnitude of 0.2 MPa and a concentration of 35% and it is notable that these findings are in keeping with the magnitudes of the affecting parameters considered in this study.

The choice of polymer can affect the production rate, fiber diameter, and energy consumption of the process, which can in turn impact the quality and cost of the final product. An increase in concentration or viscosity of PEO and PVP solutions subsequently increased the diameter of the fibers produced and production rate, along with an increase in total energy consumption to manufacture these fibers. An increase in applied pressure increased production rate and a decrease in fiber diameter. Improvements in the effects of the application of increasing pressure on energy consumption cannot be legitimately concluded for both PEO and PVP. However, energy consumption was seen to be lower with the application of 0.1 MPa of pressure, relative to no applied pressure. There may be potential for higher magnitudes of applied pressure over 0.2 MPa to improve energy consumption in the production of PEO and PVP fibers. Promoting energy efficiency was seen to be an important parameter in the principles of both Green Engineering and Green Chemistry. It is acknowledged that it is a significant challenge to incorporate all principles, however, practicing all 12 Green Chemistry and Green Engineering principles must become an aspiration for all polymer scientists and engineers. In this way, the inevitable and necessary transformation of polymer production toward a more sustainable future will be significantly supplemented.

## Conflict of Interest

The authors declare no conflict of interest.

## Data Availability

The data that support the findings of this study are available from the corresponding author upon reasonable request.
